# Behavioural adaptations of argulid parasites (Crustacea: Branchiura) to major challenges in their life cycle

**DOI:** 10.1186/s13071-015-1005-0

**Published:** 2015-07-25

**Authors:** V.N. Mikheev, A.F. Pasternak, E.T. Valtonen

**Affiliations:** Institute of Ecology and Evolution, Russian Academy of Sciences, 33 Leninskii pr, 119071 Moscow, Russia; Institute of Oceanology, Russian Academy of Sciences, 36 Nakhimovskii pr, 117997 Moscow, Russia; Department of Biological and Environmental Science, University of Jyväskylä, PL 35, 40351 Jyväskylä, Finland

**Keywords:** Fish ectoparasites, Argulus foliaceus, Argulus coregoni, Aggregative behaviour, Host searching, Behavioural tactics, Vectors

## Abstract

Fish lice (*Argulus* spp.) are obligate ectoparasites, which contrary to most aquatic parasites, retain the ability to swim freely throughout the whole of their life. In fish farms, they can quickly increase in numbers and without effective control cause argulosis, which results in the reduced growth and survival of their fish hosts. The morphology of *Argulus* spp, including their sensory organs, is suitable for both parasitism and free-swimming. By spending a considerable amount of time away from their host, these parasites risk being excessively dispersed, which could endanger mating success. Here we present a review of recent studies on the behaviour of *Argulus* spp, especially the aggregative behaviour that mitigates the dilution of the parasite population. Aggregation of parasites, which is especially important during the period of reproduction, occurs on different scales and involves both the aggregation of the host and the aggregation of the parasites on the host. The main behavioural adaptations of *Argulus* spp, including searches for hosts and mates, host manipulation and host choice, are all focused on the fish. As these ectoparasites repeatedly change hosts and inflict skin damage, they can act as vectors for fish pathogens. The development of environmentally friendly measures for the control and prevention of argulosis needs to take into account the behaviour of the parasites.

## Introduction

Although the behaviour of parasites has attracted serious attention [1–4], it is still little studied when compared with their morphology and physiology. The high abundance of descendants and the efficient exploitation of hosts are widely accepted as the main determinants of parasite fitness [5, 6]. Despite the close association with their host, all macroparasites have dispersal stages, which help to expand the parasite’s range, find hosts and facilitate genetic exchange. Heteroxenous parasites have to change hosts several times in their life history, whereas monoxenous forms are able to complete their life cycle on one host, as in the case of most parasitic copepods. Under such a strategy, the role of a free-swimming stage is important only for the short period of dispersal.

However, there is a group of obligate aquatic ectoparasites of the subclass Branchiura, which also retain the ability to swim freely at the adult stage. The most diverse, abundant and widely distributed group of the Branchiura are species of the genus *Argulus* (fish lice), which parasitize fishes [7, 8]. Species of this genus inhabit mainly freshwaters from the Tropics to the Holarctic. The behavioural complex of argulids, from larvae to mature adults, includes behaviour on the host and behaviour when free-swimming. We will focus on the behaviour of *Argulus* spp. when free-swimming and, later on, address the term ‘behaviour’ to this part of the behavioural complex.

Swimming *Argulus* spp. are easily dispersed, both by water currents and their own activity. Parasite dispersal associated with water movements has received much attention in studies on sea lice [9]. Dispersal may decrease the local density of parasites and hamper mate finding. In boreal waters, where the density of fish and parasite populations is often low, this could seriously reduce the reproduction success of the parasite population. To increase encounters between parasites and their fish hosts and between parasite males and females, the dispersal of the fish lice should be counterbalanced by behaviour leading to their aggregation. The necessity to form aggregations makes the behaviour, especially movement and orientation, a pivotal aspect of the biology of *Argulus* spp. Such an aggregation could occur on the fish hosts, on various interfaces or via an attraction to each other in the water column.

Successful host searching tactics of free-swimming *Argulus* spp. needs to include adjustments to changing behaviour of their target fish. Are these parasites choosy or non-selective in relation to different fish species? Do they change their tactics according to diurnal changes in fish behaviour? Can monoxenous argulids manipulate their fish hosts as some trophically transmitted heteroxenous parasites do [10–12]? When parasites have matured and are ready to reproduce the main challenge is to find a mate. Aggregation of both parasites and hosts would significantly facilitate a search for a mate. Are males and females different in their mate searching behaviour? What behaviours lead to an aggregated distribution of fish lice?

Despite our basic knowledge of simple behavioural responses and the main sensory modalities employed by free-swimming fish lice [13–15] and a recent book on biology and behaviour of ectoparasites [16], we are still far from understanding the adaptations that enable fish lice to find both hosts and mates and maintain sustainable populations. A programme of experimental and field studies on the behaviour of *Argulus foliaceus* and *A. coregoni* has been carried out in Central Finland from 1997 onwards [17–23]. These two species are widely distributed in Eurasia and cause epizootics at fish farms [24]. Even more important could be knowledge of the role of argulids in the transmission of pathogens, such as bacteria, viruses and fungi, between fish.

The present paper aims to review recent findings on key aspects of fish lice behaviour when free-swimming, including the coordinated use of various sensory organs, and parasite movement and selective responses in relation to the hosts and conspecifics. We expect that knowledge of these aspects will help us understand how argulids meet the major challenges faced during their life history, to create measures against epizootics and to draw attention to their role as vectors of fish pathogens.

## Review

### Main sensory modalities and host searching tactics of fish lice

The first experimental study focusing on responses of *A. foliaceus* to environmental and host-induced stimuli was carried out by Konrad Herter [13], who concluded that this parasite encountered fish randomly. Surprisingly, *A. foliaceus* rarely reacted to the fish swimming as close as 3–5 cm from the parasite. Only closer contact ended with an attachment. It was shown that *A. foliaceus*, when close to the fish, responded primarily to water movements, chemical and tactile stimuli [13–15]. Vision was regarded as unimportant for host searching and served merely to discriminate between light and dark parts of the experimental tank [13].

A random encounter of hosts as the main mechanism for host finding is doubtful because the movement of small animals in a viscous medium is costly and extremely ineffective [25]. As to the role of vision, the large, well-developed, faceted eyes of *A. foliaceus* [26] suggest that vision is used for more than just for discriminating between light and dark. We repeated experiments on the host searching behaviour of *A. foliaceus* in glass aquaria similar to those used by Herter, and obtained virtually the same results [17]. Then, the experiment was repeated in aquaria with dark, non-reflective walls, which did not produce spurious visual targets. This simple change in experimental conditions resulted in a dramatic change in the swimming pattern and host searching efficiency of *A. foliaceus* [17]. Their swimming speed decreased by a factor of 4–5, but the rate of attachment increased by almost a factor of 10. We observed directional movements of 10–15 cm towards highly reflective objects and the attraction of *A. foliaceus* to most reflective fish [17, 18]. In glass aquaria, the parasites appeared confused by numerous light spots, which impaired the use of vision.

The behaviour of *A. foliaceus* was different in the dark and light: two alternative host searching tactics were found (Table 1). In daylight, the parasite employed “hover-and-wait” tactics with low swimming speed and an inclined position of the body. In the dark, “cruising” tactics were employed, characterized by a much higher swimming speed and a horizontal position of the body [18]. Vision, olfaction and mechanoreception are used in daylight, whereas only the latter two are used at night (Table 1). Swimming speed was 5–6 times greater at night than in the daylight. Within both dark and light periods, swimming was controlled by both the host-induced (visual, chemical and hydromechanical) and internal (state of hunger) factors. Host-induced cues increased mean swimming speed by a factor of 1.5–3 [18]. In *A. foliaceus* starved for 1–2 days, the swimming speed was 3–4 times greater than that of fish lice freshly detached from the host. A longer starvation caused a decrease in swimming activity [18].

**Table 1:** Fish lice are efficient host searchers, both day and night

Behavioural traits	Period	
	Day	Night
Sensory modalities	Vision Olfaction Mechanoreception	Olfaction Mechanoreception
Motor activity	Low	High
Internal modifiers of activity	Hunger state	Hunger state
Fish induced stimuli	Visual Chemical Mechanical	Chemical Mechanical
Host searching tactics	Hover-and-wait	Cruising
	
More efficient at host searching	*Argulus coregoni*	*Argulus foliaceus*

Most quantitative estimations were obtained for *Argulus foliaceus* [17, 18]. Experimental data on olfaction, vision and host searching for *A. coregoni* [19, 20] are included

With regard to changes in the use of the sensory organs during the ontogeny of fish lice, newly hatched larvae and early juveniles of *A. coregoni* were attracted by and reacted indiscriminately to every bright object. Among the test fishes they chose those species with highest reflectivity of the body (i.e. cyprinids, with silvery sides, compared to darker salmonids) [19]. The relative roles of vision and olfaction in host recognition changed with the maturation of parasites. From the age of about 3 weeks, *A. coregoni* did not respond to bright objects in the absence of a concomitant chemical stimulation, such as salmonid fish odour [19]. At this stage, *A. coregoni* had developed a strong preference for salmonid fishes [20]. The innate and ecological aspects of such preference will be discussed below.

### Argulus-fish behavioural interactions. Can fish lice manipulate their hosts?

The host searching success of argulids depends not only on their behaviour and sensory abilities but also on host availability. We found that diurnal changes in the swimming patterns of juvenile perch (*Perca fluviatilis*) and roach (*Rutilus rutilus*) influenced the attachment rate of *A. foliaceus*. At night, perch move slowly and intermittently, which facilitates host searching for the parasite. Faster and continuously swimming roach were a less available target [17]. In the daytime, when both fishes moved continuously, the more reflective roach attracted significantly more parasites than perch [17]. The behaviour of *A. foliaceus* is well adjusted to the diurnal variation in the behaviour of the host: in the daytime, hover-and-wait tactics are used to intercept and attach to a nearby swimming fish; at night, cruising tactics are used to find and attach to slowly moving or stationary fish [18]. Increased nocturnal activity resulted in an increase in energy expenditure by more than 25 % [18]. Free-swimming fish lice can survive outside the host for no longer than several days [14, 27]. At a low mean host density, these parasites would benefit if the fish were aggregated. Can *Argulus* spp. manipulate their host’s behaviour to make them more available?

Two types of parasite-induced change in fish behaviour can be considered as host manipulation. A short-term effect (seconds to tens of seconds) is related to behavioural responses of an individual fish (jerking, vigorous turning) to the attachment of *A. canadensis* [28] and *A. foliaceus* [29]. Such behaviour enhances visual, olfactory and mechanical stimulation, attracting more parasites to the already infected fish [30]. This results in an aggregated distribution of parasites among their hosts [28]. A similar aggregating mechanism has been observed in another ectoparasite-host system, i.e. copepods on brook trout fry [31]. Another type includes longer term effects (minutes to tens of minutes). The attachment of *A. foliaceus* to juvenile rainbow trout (*Oncorhynchus mykiss*) made them swim closer to each other, reducing swimming speed and aggression [29]. The consolidation of fish shoals and slower movement facilitates the transmission of parasites. We suggest that changes in fish behaviour are triggered by the release of an alarm substance from fish skin damaged by attached parasites. The formation of tight shoals is similar to the defence behaviour which fish exhibit when a conspecific fish is injured by a predator [32, 33]. We suggest that, in order to manipulate their hosts, parasites exploit this mechanism, which evolved in fish as an antipredatory behaviour [29].

Could other organisms influence behaviour of fish and *Argulus* spp. in a manipulatory way? If a pathogen uses *Argulus* spp. as vectors within the fish-*Argulus* system, it can influence characteristics of both the fish and the parasite, facilitating its own transmission. Fish infected with *Flavobacterium columnare* may develop columnaris disease with the symptoms of erosion and necrosis of the gills and skin, usually around the dorsal fin [34]. These injuries likely cause changes in any olfactory cues produced and in fish activity. Weakened fish reduce their swimming behaviour, which facilitates the attachment of *A. foliaceus* [18]. Bacteria may also impact on the behaviour of the hosts and vectors [35]. Olfactory cues signalling the presence of a debilitated fish might help the parasite in finding such a fish. Debilitated fish infected with *Saprolegnia* spp. carried much greater numbers of argulids compared with fish without a fungal infection (our unpublished data). This suggests that the host microbiota may affect attractiveness of hosts to vectors [36].

Vector competence is the ability of a vector to transmit a pathogen, i.e. “the intrinsic permissiveness of a vector to be infected, to replicate and to transmit a pathogen” [37]. Could a pathogen influence the frequency of host change by *Argulus* spp. to facilitate the transmission of a pathogen? Such a manipulation would be expected to focus on *Argulus* spp. males, because they are the most mobile members of the population. It has been said that many micro-organisms could be more powerful modifiers of their host’s biology and behaviour than macroparasites [35, 38]. Adaptive modifications of behaviour of both *Argulus* spp and fish in a host-parasite-pathogen system require further studies.

### Host specificity. Why *Argulus coregoni* is more choosy than *A. foliaceus*?

*Argulus coregoni* and the smaller *A. foliaceus* co-exist in the lakes and rivers of Central Finland. The distribution of these parasites among fish species is different. *A. foliaceus* is opportunistic and can be found on any fish species, with the highest abundance on perch, roach and pike [21, 39], whereas *A. coregoni* is a specialist on salmonids [40, 41]. However, no host specificity was found during the early ontogeny of *A. coregoni* [19, 20]. Metanauplii and early juveniles attach to every available fish, preferring the most reflective targets [19]. Host specificity developed with maturation, beginning at the pre-adult stage [20]. Increasing preference for salmonids was accompanied by an enhanced rate of detachments of fish lice, especially males, from the hosts [42, 43]. By the spawning period, most *A. coregoni* had shifted to salmonids [20].

**Fig. 1:**
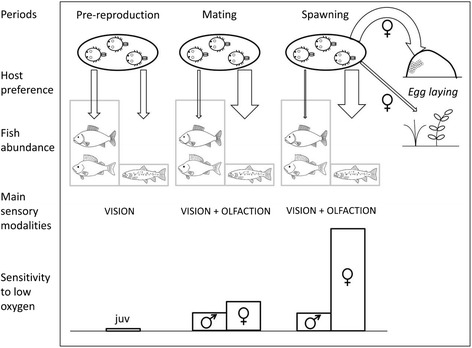
Behavioural and physiological basis of host specificity and habitat fidelity in *Argulus coregoni.* Before the reproductive period, juveniles attach to any available fish. Vision is the main sensory modality at this stage. A strong preference for salmonid fishes (*widest down arrow*) develops during the mating period. At this stage, vision is supplemented with olfaction. The concentration of adult parasites on oxyphyllic salmonids keeps the population of *A. coregoni* within well oxygenated habitats where stony bottoms are suitable for egg laying. The sensitivity to low oxygen (greatest in mature females) was assessed as the mortality rate at 10 % saturation

Why do these co-existing argulid species differ in their host preference? *A. coregoni* adults are much larger than those of *A. foliaceus*. Body length in mature *A. coregoni* females is 13–15 mm [40], whereas that of *A. foliaceus* is about 5–7 mm [15]. The large size of *A. coregoni* is beneficial in terms of finding and attaching to large, agile salmonid fishes. On the other hand, the ratio of surface area to body volume in *A. coregoni* is about half that of *A. foliaceus*, which makes the former more sensitive to low oxygen concentration [20]. Gravid females are especially vulnerable to oxygen deficiency (Fig. 1) [20]. We consider that an increasing preference for salmonids in maturing *A. coregoni* helps them to remain in well oxygenated habitats.

The preference of *A. coregoni* for salmonids is innate and not acquired through habituation to the hosts to which the newly hatched larvae attach [20]. *A. coregoni*, reared on both cyprinids and salmonids, first exhibited a pronounced preference for salmonids when they reached the length of 4–5 mm [20]. However, this species retained the ability to develop, mature and spawn when only cyprinid hosts were available [22]. The ability of argulids to change hosts mitigates the difficult task of finding an appropriate host and habitat. Although the fitness of *A. coregoni* on non-salmonid hosts is lower than on salmonids (due to the more frequent detachments from non-salmonids and thus the shorter period of residence on them), these hosts could maintain the parasite population in cases of a sudden drop in the salmonid population.

Comparison between the two *Argulus* species emphasizes the role of body size as an important determinant of host searching behaviour, specificity and habitat preference in these parasites. The role of body size as a determinant of crucial physiological processes and their ecological effects is well known [25]. For closely related obligate parasite species of different sizes, size-based difference in behaviour and ecology was shown for the first time [19, 20]. Large size is beneficial for host searching and attachment in running and turbulent waters, but it also imposes physiological and ecological limitations, restricting the distribution of *A. coregoni* within a water system. The difference in the preferred habitats of the two co-existing argulids is reflected in the relative importance of sensory organs. Vision is more important for *A. coregoni*, because olfaction and mechanoreception are of limited efficiency in their preferable habitats. The highest infection rate was recorded for *A. coregoni* during daylight, whereas, for *A. foliaceus*, it was during the dark [20]. On the other hand, *A. foliaceus*, which mostly inhabits still and turbid shallow waters, relies more on olfaction and mechanoreception.

### Reproduction and behaviour. Division of labour between males and females

The aggregation of fish lice on fishes is a prerequisite for their successful mating [29], because even mature parasites fail to react to each other when swimming in water. Parasites on a heavily infected fish have no problems finding a conspecific specimen of the appropriate sex and state of maturity. If there are no appropriate mates on a host, the parasites must leave this host. Males of the two species play a leading role in mate searching. They frequently detach from the hosts (Table 2) and spend much more time swimming than females [21, 43]. Males of *A. coregoni* are able to detect and respond to sex pheromones produced by females attached to a fish [44]. We have observed that mature females of *A. foliaceus* swimming in the water do not attract males. Even at high concentrations (10 ind l^−1^), adult males do not react to females in water. Thus, the host provides *Argulus* spp. not only with food and substrate but also acts as a meeting point, provides transportation and triggers the stimuli that facilitate sexual interactions.

**Table 2:** Reproduction, behaviour, sexual dimorphism and sex ratio of *Argulus coregoni* and *A. foliaceus*

Period of the life cycle	Behaviour		Body size	Sex ratio
	Males	Females		
Juveniles	Strongly attached to host	Strongly attached to host	Males larger	Males more abundant
Pre-adults	Moderate rate of detachment	Almost no detachments	Similar size	Similar abundance
Adults	High rate of detachment - for mate searching	Low rate of detachment - for egg-laying	Females larger	Females more abundant

Data on body size and sex ratio of juveniles were obtained only for *A. coregoni*

Males of fish lice risk energy loss and increased mortality while swimming and switching hosts. Females of both *A. foliaceus* and *A. coregoni* are stationary on their host [21, 44] and produce pheromones [44] like pre-adult female sea lice, *Lepeophtheirus salmonis* do when attracting males [45]. Together with the fish and their ‘odour’, females make large, attractive targets for males. Behavioural dimorphism between males and females is related to size dimorphism (Table 2). As in other ectoparasitic crustaceans, e.g. copepods, adult females of *A. foliaceus* and *A. coregoni* are markedly larger than adult males [21, 42]. However, at earlier life history stages, juvenile males of *A. coregoni* are the larger. They grow faster and are more numerous than females, until they reach the pre-reproductive period. Subsequently, males become smaller and less numerous than females (Table 2) [42]. This is due to food deprivation, high energy expenditure and an elevated risk of mortality of males while searching for mates. Females feed continuously, grow faster and accumulate energy for egg production.

In *A. foliaceus* and *A. coregoni*, both sexes bear the high energy costs of reproduction. Males spend a great amount of energy searching for a mate, whereas females invest heavily in egg production. Energy is also needed to search for an appropriate substrate for spawning. In general, the role of the sexes resembles that of parasitic copepods, but fish lice females, unlike copepod females [46], retain the ability to leave their hosts and swim freely even after laying a portion of their eggs. Females of *Argulus* spp. invest precious energy in a costly spawning behaviour, providing egg clutches with the suitable conditions for incubation. In choosing the appropriate sites for egg deposition females play an important role in maintaining the population within a favourable habitat.

### Behaviour, habitat fidelity and the life cycle of fish lice

Behaviour, as mentioned above, plays a crucial role throughout the life of argulids between hatching and spawning. Besides searching for hosts and mates, behaviour is also essential in maintaining population integrity. For this purpose, parasites have to remain within restricted parts of a water body. This is particularly important at the beginning of the seasonal cycle, which starts with a mass hatching of metanauplii from the overwintered eggs. Newly hatched metanauplii must quickly find a host, so they need to be in areas of high fish abundance; these are the spawning and nursery grounds. This is guaranteed by gravid females laying their eggs either on stones (in the case of *A. coregoni*) or on vegetation in shallow littoral areas (for *A. foliaceus*). It was suggested that the location of egg laying is determined by the habitat usage of host fish [47].

Habitat fidelity and potential for aggregation in fish lice are associated not only with preferred egg laying areas of a particular species but also with ecologically distinct sets of fish hosts. *A. foliaceus* can be found on every fish species in lakes and rivers. They usually prefer fish inhabiting shallow stagnant waters, such as percids, cyprinids and esocids [39], *A. foliaceus* lays eggs on vegetation, wood debris and roots [14, 15]. This relatively small species readily tolerates low oxygen concentrations [20] that often occur in shallow waters. The larger *A. coregoni*, especially at the adult stage, strongly prefers salmonid fishes inhabiting well oxygenated running waters. Such habitats are often associated with stony bottoms, the best substrate for *A. coregoni* egg laying [48]. Females choose dark-coloured stones of medium size, preferring sites that are not exposed to strong currents. Eggs are laid not too close to the bottom, so that sediments do not cover them [23, 48].

**Fig. 2:**
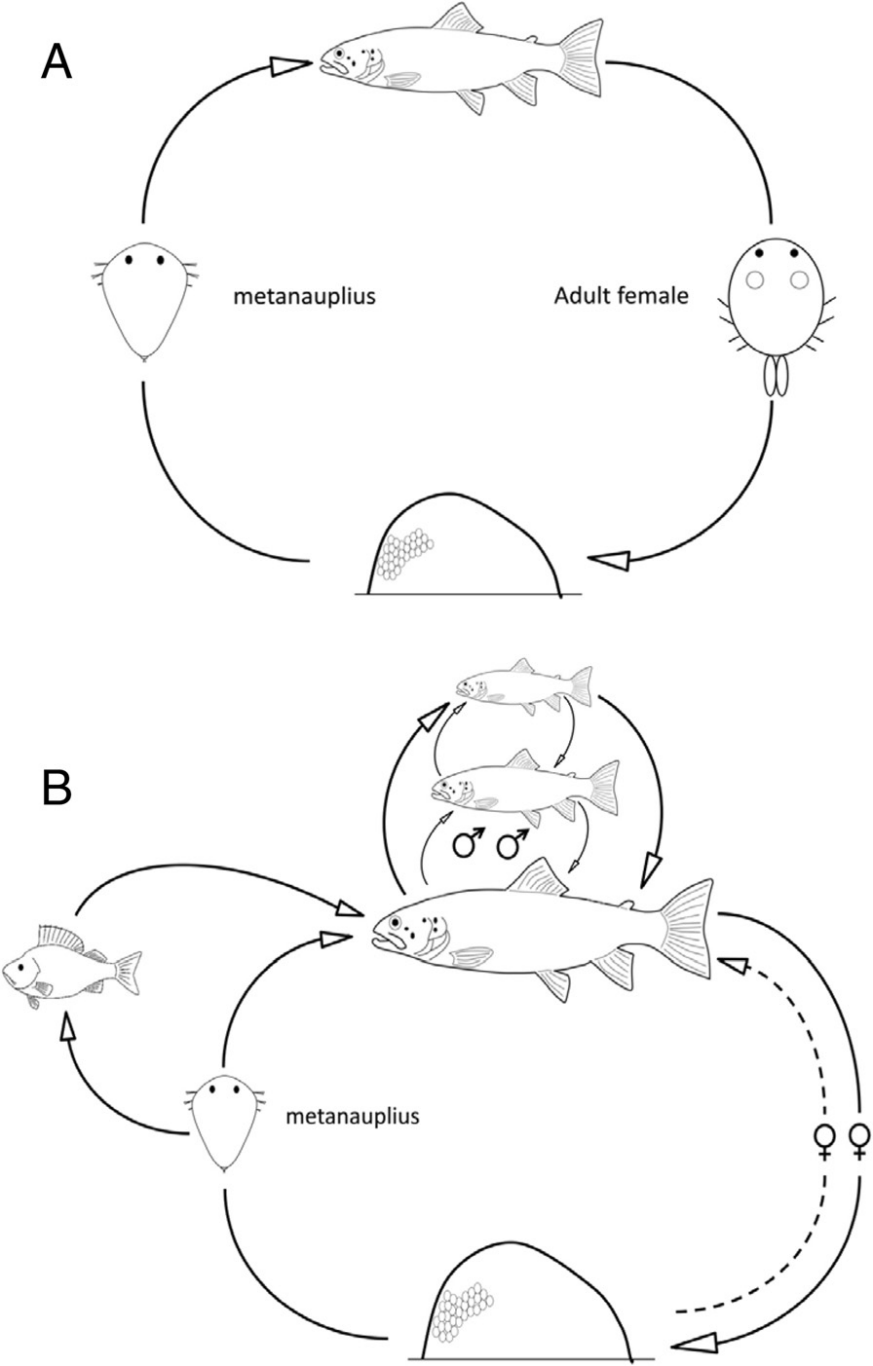
Life cycle of *Argulus coregoni*. **a** a generalized scheme showing the key stages and sites. The larva hatches from the egg and attaches to a salmonid host, where it grows to adult. Copulation occurs on the host, after which the females detach to deposit eggs on a suitable substrate. **b** diverse behavioural interactions make the life cycle more complex, with more hosts being involved. Free swimming larvae attach to any available host, grow and then switch to more suitable hosts (salmonids). Adult males, the most mobile members of the population, may transfer between many salmonid hosts in the search for a mate. Gravid females deposit eggs in several different, consecutive clutches, returning to the host in between

The life cycle of *A. coregoni* includes more interactions and links than previously believed e.g., [14, 49] (Fig. 2). Behavioural relationships between *A. coregoni* and their hosts make life trajectories of these monoxenous parasites rather diverse and variable. An individual *A. coregoni* specimen may change fish hosts many times during its life [15, 22, 43]. Metanauplii and juveniles are strongly attached and rarely abandon their host [22, 42, 43]. With approaching reproduction, the mate searching activity of males increases. They often detach from their host if appropriate mates are not available there. Females detach from their hosts to lay eggs on a suitable substrate. After laying a clutch of eggs, females have to restore their energy levels and, thus, attach to a new host in order to be able to lay the next egg clutch. For *A. coregoni*, a specialist on salmonids, there is another reason for changing hosts, i.e. finding an appropriate host. This restricts variations in life trajectories in the larger, oxygen-demanding *A. coregoni* compared with *A. foliaceus* and, probably, other argulid species, which are all smaller than *A. coregoni*.

### Potentials for management and control of fish lice populations

Ectoparasitic crustaceans can cause epizootics in relation to both marine and freshwater fish farms [9, 50–52], Ectoparasitic infections of fish, including argulosis, result in reduced growth and survival, and also increased production costs in commercial farms [53, 54]. Most of the applied methods to fight against crustacean ectoparasites are based on the use of chemicals [50, 54]. To control crustacean epizootics, and *A. coregoni* in particular, emamectin benzoate has been widely used in both marine and freshwater aquaculture [55–57]. However, sensitivity to chemical treatments eventually reduces because of habituation. As a result, the Integrated Pest Management concept (IPM) was introduced to aquaculture e.g. [58, 59]. An IPM programme includes measures to prevent infections and monitor infection levels, which requires a knowledge of the ecology and behaviour of the parasites [24, 47, 60, 61].

Prevention is fundamental to IPM, and a crucial point in preventing the mass development of fish lice is a reduction in egg abundance. A model simulating the population dynamics of *A. coregoni* [62] showed that infection levels largely rely on the number of eggs in the egg-bank [63] and destroying eggs with all available means would greatly reduce the size of a parasite population. To choose an efficient technique for the reduction of egg numbers, the species specific time of intensive spawning and the location and preferred substrate for egg-laying should be taken into account [47, 48, 61]. Two groups of measures can be distinguished: 1) depriving fish lice of suitable substrates or destroying substrates already covered with laid egg clutches; and 2) attracting gravid females to artificial substrates (egg collectors) [23, 60, 64]. The first approach implies fallowing farm sites between successive stockings [65], draining and drying ectoparasite egg laying sites and surfaces, and/or collecting stones with egg clutches [24]. Within the second approach, artificial egg collectors consisting of dark plates with rough surface are placed in suitable egg laying sites prior to the period of spawning. Most of the eggs are then laid on the underside of the plates [23], which need to be removed and cleaned several times during spawning.

The pool of eggs could also be reduced prior to spawning by shaking *A. coregoni* and *A. foliaceus* in hand nets in a water container. This simple procedure has proven to be efficient: more than 80 % of the originally attached fish lice were dislodged from the fish. Dislodgement rates of gravid females were especially pronounced. Shaking could be used on a small scale, in response to heavy infections, when other treatment options are limited, or when done in connection with normal fish husbandry practices (fish grading, vaccination, transfer etc.) [24].

To collect free swimming *A. coregoni* for research purposes, ‘light traps’ have been used [66]. The collection of unattached juveniles with such devices could be efficient, because of their strong reaction to the brightest objects [19]. Certainly, such an approach could not be recommended for the control of the fish lice infections in large farms [50], but it may be used locally as a supplementary method of reducing the number of free-swimming stages.

Attached ectoparasites, including fish lice, cause epithelial damage and evoke stress responses in fish, which may, in turn, induce secondary effects [49]. The most important of these is increased fish susceptibility to various fungal, bacterial and protozoan infections [67–71]. This could be aggravated by reduced nourishment, stunted growth and decreased host immunocompetence [53, 54, 72]. We tested whether infection with *A. coregoni* leads to a higher susceptibility of fish to a bacterial disease (*Flavobacterium columnare*) [71]. A clear effect of concomitant infection, exhibited as increased fish mortality, represented the first experimental support for the hypothesis that *Argulus* spp. can facilitate the transfer of a serious bacterial disease. No effect of concomitant infections with *F. columnare* and cercariae of *Diplostomum spathaceum* were found [73]. As compared with *D. spathaceum*, skin damage and stress caused by *A. coregoni* are more severe, which makes the latter more likely to result in the introduction of bacterial diseases [71].

The role of *Argulus* spp. as vectors and their microbiota have not yet been thoroughly studied. Most of the studies on the microbiota of the vectors and their vectorial competence have focused on the terrestrial arthropods e.g., [35, 74, 75]. Our knowledge of the behavioural interactions between fish lice and their hosts suggests that these parasites act as efficient vectors. The ability of *Argulus* spp. to reduce host resistance (mechanical damage and impaired immunity) and repeated attachment and detachment from the hosts, together with a high level of swimming activity and diverse behaviour, make these parasites potentially more efficient vectors than copepods, a more widely distributed and prolific group of ectoparasitic crustaceans. During their life history, fish lice not only switch hosts many times but also damage them, which may facilitate either the direct (from water) or indirect (via vectors) transfer of pathogens. They also cause stress each time when they attach and pierce the skin of a fish [76] and such stress, in turn, reduces immunocompetence [77, 78] and thus may facilitate the transmission of pathogens.

*Argulus* spp. possess diverse behavioural adaptations that help them in completing their life cycle. When developing measures to counteract epizootics at fish farms, one has to take into account the variety of these adaptations. This may help in the improvement of environmentally friendly measures, which in the long term, could be more efficient than chemical treatments.

## Conclusion

The laying of eggs on the substrate away from the host, and retaining the ability for free swimming throughout their entire life, means that fish lice face the risk of excessive dispersal; this complicates their main and vital tasks, i.e. to find a host and a mate. To counterbalance the risk, *Argulus* spp. use diverse behaviours resulting in aggregation. This is especially important during the period of reproduction (searching for mates), when males have to find both food and a mate. It is not only the more mobile males but also the females who contribute, in the form of pheromone production, to aggregative mechanisms. Even gravid females benefit from aggregation when they have to return to the host after laying a clutch of eggs. A modified behaviour and physiology of the fish induced by the attached parasites facilitates their finding of, and attachment to, conspecific hosts. We found no evidence for the aggregation of fish lice away from the host. This means that host searching is the pivotal activity in the behavioural repertoire of these parasites. Aggregation occurs on different scales and involves both the aggregation of fish (a host manipulation effect) and the aggregation of parasites on a fish. They can also gather in certain areas of the fish surface. Aggregation is particularly important in cold temperate waters, where the density of both fish and fish lice is usually low. In order to increase the success of host searching, *A. foliaceus* and *A. coregoni* are active day and night, employing all of their sensory organs; use alternative behavioural tactics adjusted to temporal variations in fish activity; and modify fish behaviour to make them more available for free swimming parasites. With such diverse and well-developed sensory equipment and behaviour, *Argulus* spp. can quickly increase their population density in fish farms and cause serious problems when effective control measures are lacking. These problems are related not only to the direct harmful effects of the ectoparasites but also to indirect effects when argulids can act as vectors for pathogens. An increased knowledge of the microbiota is needed to understand whether fish pathogens can replicate within the body of *Argulus* spp. and influence their behaviour and physiology, thus enhancing transmission success.
